# Construction of Binary Quantum Error-Correcting Codes from Orthogonal Array

**DOI:** 10.3390/e24071000

**Published:** 2022-07-19

**Authors:** Shanqi Pang, Hanxiao Xu, Mengqian Chen

**Affiliations:** College of Mathematics and Information Science, Henan Normal University, Xinxiang 453007, China; dm48xuhanxiao@163.com (H.X.); chenmengqian236@163.com (M.C.)

**Keywords:** quantum error-correcting codes, *k*-uniform states, orthogonal array, orthogonal partition

## Abstract

By using difference schemes, orthogonal partitions and a replacement method, some new methods to construct pure quantum error-correcting codes are provided from orthogonal arrays. As an application of these methods, we construct several infinite series of quantum error-correcting codes including some optimal ones. Compared with the existing binary quantum codes, more new codes can be constructed, which have a lower number of terms (i.e., the number of computational basis states) for each of their basis states.

## 1. Introduction

Errors are inevitable in quantum information processing [1], so quantum error-correcting codes (QECCs) are very important for quantum communication and quantum computing. In 1995, Shor [1] gave the simplest quantum simulation of a classical coding plan and then constructed the first QECC. In 1998, Calderbank et al. provided a close connection between QECCs and classical error correction codes [2], which leads to constructing QECCs from known classical error correction codes. In recent years, the research on QECCs especially on binary QECCs has made great progress. Feng and Ma made a way to obtain good pure stabilizer quantum codes, binary or nonbinary [3]. Li and Li obtained quantum codes of minimum distance three which are optimal or near optimal, and some quantum codes of minimum distance four which are better than previously known codes [4]. Feng and Xing presented a characterization of (binary and non-binary) quantum codes. Based on this characterization, they derived a method to construct pure *p*-ary quantum codes with dimensions not necessarily equal to powers of *p* [5]. Some other constructions of non-stabilizer codes, such as CWS codes [6], the codes in [7], and permutation-invariant codes such as in [8,9,10,11] have been studied. However, the majority of binary QECCs constructed so far are stabilizer codes [12,13,14]. The main goal of this work is to link between orthogonal arrays and binary QECCs and to construct more families of new codes.

Orthogonal arrays (OAs) play a more and more important role in quantum information theory [15,16,17,18,19,20,21,22]. An r×N array *A* with entries from a set S={0,1,…,s−1} is said to be an orthogonal array with *s* levels, strength *t* (for some *t* in the range 0≤t≤N) if every r×t subarray of *A* contains each *t*-tuple based on *S* as a row with the same frequency. We will denote such an array by OA(r,N,s,t). Recently, many new methods of constructing OAs, especially high strength OAs, have been presented, and many new classes of OAs have been obtained [23,24,25,26,27,28,29,30,31,32,33]. An OA(r,N,s,t) is said to be an irredundant orthogonal array (IrOA) if, in any r×(N−t) subarray, all of its rows are different [18]. A link between an IrOA with *d* levels and a *t*-uniform state was established by Goyeneche et al. [18], i.e., every column and every row of the array correspond to a particular qudit and a linear term of the state, respectively.

**Connection** **1** ([18]). *If* $L=\left(\begin{array}{cccc} s_{1}^{1} & s_{2}^{1} & \cdots & s_{N}^{1}\\ s_{1}^{2} & s_{2}^{2} & \cdots & s_{N}^{2}\\ \vdots & \vdots & \cdots & \vdots \\ s_{1}^{r} & s_{2}^{r} & \cdots & s_{N}^{r} \end{array}\right)$ *is an* IrOA(r,N,s,t), *then the superposition of r product states*,

$$|\Phi \rangle =\frac{1}{\sqrt{r}}(|s_{1}^{1}s_{2}^{1}\ldots s_{N}^{1}\rangle +|s_{1}^{2}s_{2}^{2}\ldots s_{N}^{2}\rangle +\cdots +|s_{1}^{r}s_{2}^{r}\ldots s_{N}^{r}\rangle )$$

*is a t-uniform state*.

More and more attention has been paid to the construction and characterization of *t*-uniform states from OAs [15,16,17,18,34,35,36,37,38,39]. Very interestingly, uniform states are closely related to QECCs. Goyeneche and Życzkowski stated ${\left(\left(N,1,k+1\right)\right)}_{d}$ QECCs are one-to-one connected to *k*-uniform states of *N* qudits [18]. Shi et al. also presented the relation between a pure QECC and *t*-uniform state [40]. It is these new developments in OAs and uniform states that raise the possibility of constructing QECCs from OAs.

In this paper, the Hamming distance and minimal distance (MD) of OAs are applied to the theory of quantum information. By using difference schemes, orthogonal partitions and a replacement method, some new methods to construct pure quantum error-correcting codes are provided from orthogonal arrays. As an application of these methods, we construct several infinite series of quantum error-correcting codes including some optimal ones. Compared with the corresponding binary quantum error-correcting codes in [12,41], more new codes can be constructed, which have fewer terms for each of their basis states.

## 2. Preliminaries

First, the following concepts and lemmas are needed.

Let $A^{T}$ be the transposition of matrix *A* and $\left(2\right)={\left(0,1\right)}^{T}$. Let $0_{r}$ and $1_{r}$ denote the r×1 vectors of 0s and 1s, respectively. If $A={\left(a_{ij}\right)}_{m\times n}$ and $B={\left(b_{ij}\right)}_{u\times v}$ with elements from a Galois field with binary operations (+ and ·), the Kronecker product A⊗B is defined as $A⊗B={\left(a_{ij}\cdot B\right)}_{mu\times nv}$, where $a_{ij}\cdot B$ represents the u×v matrix with entries $a_{ij}\cdot b_{rs}$(1≤r≤u,1≤s≤v), and the Kronecker sum A⊕B is defined as $A⊕B={\left(a_{ij}+B\right)}_{mu\times nv}$ where $a_{ij}+B$ represents the u×v matrix with entries $a_{ij}+b_{rs}$(1≤r≤u,1≤s≤v) [23,24]. Let ${\left(C^{2}\right)}^{⊗N}=\underset{N}{\underset{︸}{C^{2}⊗C^{2}⊗\cdots ⊗C^{2}}}$. Let $Z_{2}^{N}=\underset{N}{\underset{︸}{Z_{2}\times Z_{2}\times \cdots \times Z_{2}}}$ over ring $Z_{2}=\left\{0,1\right\}$.

A matrix *A* can often be identified with a set of its row vectors if necessary.

**Definition** **1** ([26]). *Let A be an OA(r,N,s,t) and $\left\{A_{1},A_{2},\ldots ,A_{u}\right\}$ be a set of orthogonal arrays $OA(\frac{r}{u},N,s,t_{1})$ with $t_{1}\geq 0$. If $\overset{u}{\underset{i=1}{⋃}}A_{i}=A$ and $A_{i}⋂A_{j}=\emptyset$ for i≠j, then $\left\{A_{1},A_{2},\ldots ,A_{u}\right\}$ is said to be an orthogonal partition of strength $t_{1}$ of A.*

Let A be an abelian group of order *s*. $A^{t}$, t≥1, denotes the additive group of order $s^{t}$ consisting of all *t*-tuples of entries from A with the usual vector addition as the binary operation. Let $A_{0}^{t}=\left\{\left(x_{1},\ldots ,x_{t}\right):x_{1}=\cdots =x_{t}\in A\right\}$. Then, $A_{0}^{t}$ is a subgroup of $A^{t}$ of order *s*, and its cosets will be denoted by $A_{i}^{t}$, $i=1,\ldots ,s^{t-1}-1$.

**Definition** **2** ([42]). *An m×n matrix D based on A is called a difference scheme of strength t if, for every m×t submatrix, each set $A_{i}^{t}$, $i=0,1,\ldots ,s^{t-1}-1$, is represented equally often when the rows of the submatrix are viewed as elements of $A^{t}$. Such a matrix is denoted by $D_{t}\left(m,n,s\right)$. When t=2, $D_{t}\left(m,n,s\right)$ is written as D(m,n,s).*

**Definition** **3.** *Let D be a difference scheme $D_{t}\left(m,n,s\right)$ and $\left\{D_{1},D_{2},\ldots ,D_{u}\right\}$ be a set of difference schemes $D_{t_{1}}\left(\frac{m}{u},n,s,\right)$ with $t_{1}\geq 0$. If $\overset{u}{\underset{i=1}{⋃}}D_{i}=D$ and $D_{i}⋂D_{j}=\emptyset$ for i≠j, then $\left\{D_{1},D_{2},\ldots ,D_{u}\right\}$ is said to be a partition of strength $t_{1}$ of D.*

**Definition** **4** ([42]). *Let $S^{l}=\left\{\left(v_{1},\ldots ,v_{l}\right)|v_{i}\in S,i=1,2,\ldots ,l\right\}$. The Hamming distance *HD*(u,v) between two vectors $u=(u_{1},\ldots ,u_{l})$, $v=(v_{1},\ldots ,v_{l})$ in $S^{l}$ is defined as the number of positions in which they differ. The minimal distance MD(A) of a matrix A is defined to be the minimal Hamming distance between its distinct rows.*

**Definition** **5** ([43]). *(quantum Singleton bound) Let Q be an ${\left(\left(N,K,d\right)\right)}_{s}$ QECC. If K>1, then $K\leq s^{N-2d+2}$. A QECC that achieves the equality is said to be optimal.*

**Lemma** **1** ([42]). *If s≤t and t is odd, then there exists a difference scheme $D_{t}\left(s^{t-1},t+1,s\right)$ on S.*

**Lemma** **2** ([37]). *The minimal distance of an *OA*$\left(s^{t},N,s,t\right)$ is N−t+1 for s≥2 and t≥1.*

**Lemma** **3** ([40]). *Let Q be a subspace of ${\left(C^{s}\right)}^{⊗N}$. If Q is an ${\left(\left(N,K,d\right)\right)}_{s}$ QECC, then for any (d−1) parties, the reductions of all states in Q to the (d−1) parties are identical. The converse is true. Further, if Q is pure, then any state in Q is a (d−1)-uniform state. The converse is also true.*

Lemma 3 can also be viewed as the definition of a QECC. *Q* is denoted as ${\left(\left(N,K,d\right)\right)}_{s}$, where *N* is the length of the code, *K* is the dimension of the encoding state, *d* is the minimum Hamming distance, and *s* is the alphabet size. When s=2, it is simply written as ((N,K,d)).

**Lemma** **4** ([44]). *(1) Let D be a difference matrix $D_{t}\left(m,n,s\right)$ and L be an OA(r,N,s,t) for t=2,3. Then D⊕L is an OA(mr,nN,s,t);*

*(2) Let D be a difference matrix $D_{t}\left(m,n,s\right)$ with t≥2. Then D⊕(s) is an OA(ms,n,s,t).*

**Lemma** **5** ([36]). *(Expansive replacement method). Suppose A is an OA of strength t with column 1 having s levels and that B also is an OA of strength t with s rows. After making a one-to-one mapping between the levels of column 1 in A and the rows of B, if each level of column 1 in A is replaced by the corresponding row from B, we can obtain an OA of strength t.*

**Lemma** **6** ([42]). *If s≥2 is a prime power then an *OA*$\left(s^{t},s+1,s,t\right)$ of index unity exists whenever s≥t−1≥0.*

## 3. Main Results

This section presents some new methods for the construction of QECCs. We begin with a link between OAs and QECCs. There exists a perfect match between the parameters of an OA(r,N,s,t), *A*, with an orthogonal partition $\left\{A_{1},A_{2},\ldots ,A_{K}\right\}$ of strength $t_{1}$ and the parameters of an ${\left(\left(N,K,d\right)\right)}_{s}$ QECC, which is listed in Table 1.

The construction method for a QECC *Q* with parameter ((N,K,d)) is summarized in the following Algorithm 1.
**Algorithm 1** (OA-QECCs method) OA algorithm for construction of binary QECCs.Step 1. Find an OA(r,N,2,t) with minimal distance $d^{\prime }$ and an orthogonal partition $\left\{A_{1},A_{2},\ldots ,A_{K}\right\}$ of strength $t_{1}$ by a difference scheme or a space $Z_{2}^{N}$; Step 2. Let $d=min\{d^{\prime },t_{1}+1\}$. Give logical codewords $\varphi_{1},\ldots ,\varphi_{K}$, where $\varphi_{i}$ is a (d−1)-uniform state, by $A_{1},A_{2},\ldots ,A_{K}$ and Connection 1 in the Introduction; Step 3. $\left\{\varphi_{1},\ldots ,\varphi_{K}\right\}$ can be used as a base to form the QECC Q=((N,K,d)).

**Theorem** **1.** *If t≥2 and t is odd, then we can construct a ((t+1,K,2)) QECC for any integer $1\leq K\leq 2^{t-1}$ including an optimal $\left(\left(t+1,2^{t-1},2\right)\right)$ code.*

**Proof.**  Step 1. Find an OA *A* with minimal distance $d^{\prime }$ and an orthogonal partition $\left\{A_{1},\ldots ,A_{K}\right\}$ of strength $t_{1}$ by a difference scheme.

By Lemma 1, a difference scheme $D=D_{t}\left(2^{t-1},t+1,2\right)$ exists for any odd integer t≥2. Take A=D⊕(2). Due to Lemma 4, *A* is an OA$\left(2^{t},t+1,2,t\right)$. Let $D=\left(\begin{array}{c} d_{1}\\ d_{2}\\ \vdots \\ d_{2^{t-1}} \end{array}\right)$. Then $A_{i}=d_{i}⊕\left(2\right)$ is also an IrOA(2,t+1,2,1) for $i=1,2,\ldots ,2^{t-1}$. It follows from Lemma 2 that MD(A)=2 and $\mathrm{MD}(A_{i})=t+1$;

Step 2. Let $d=min\{d^{\prime },t_{1}+1\}$. Give logical codewords $\varphi_{1},\ldots ,\varphi_{K}$, where $\varphi_{i}$ is a (d−1)-uniform state, generated by $A_{1},A_{2},\ldots ,A_{K}$ and Connection 1 in the Introduction.

Let $K=2^{t-1}$. By the relation between irredundant orthogonal arrays and uniform states (Connection 1), $\left\{A_{1},A_{2},\ldots ,A_{2^{t-1}}\right\}$ can generate $2^{t-1}$ one-uniform states $\left\{\varphi_{1},\varphi_{2},\ldots ,\varphi_{2^{t-1}}\right\}$;

Step 3. The uniform states $\varphi_{1},\ldots ,\varphi_{K}$ are just the logical codewords of a QECC $Q=(\left(t+1,2^{t-1},2\right))$.

By Lemma 3 and Definition 5, *Q* is an optimal code.

Furthermore, if we take $Q_{K}$ to be the subspace spanned by $\left\{\varphi_{1},\ldots ,\varphi_{K}\right\}$ for integer $1\leq K\leq 2^{t-1}-1$, then it is a ((t+1,K,2)) code.

In particular, for t=1, taking $|\varphi \rangle =\frac{1}{\sqrt{2}}(|00\rangle +\left|11\rangle \right)$ as a basis state, we have a ((2,1,2)) QECC.

Compared with the binary QECCs in [12], the ((N,K,2)) QECCs obtained from Theorem 1 for N=4,6,8 have fewer terms for each basis state and more dimensions *K* not necessarily equal to powers of 2. The comparison is put in Table 2, where “*K*” denotes the dimension of QECCs and “No.” represents the number of terms for each basis state.

The following is about construction of QECCs with odd length *N* and minimum distance 2. □

**Theorem 2.**  *(1) When N≡1(mod4), we can construct an ((N,K,2)) QECC with $K=1+C_{N}^{2}+C_{N}^{4}+\cdots +C_{N}^{\frac{N-5}{2}}+C_{N-1}^{\frac{N-3}{2}}$;*

*(2) When N≡3(mod4), there exists an ((N,K,2)) QECC with $K=1+C_{N}^{2}+C_{N}^{4}+\cdots +C_{N}^{\frac{N-3}{2}}$.*

**Proof.**  (1) $Z_{2}^{N}$ has $C_{N}^{0}$ vectors with weight 0, $C_{N}^{2}$ vectors with weight 2, $C_{N}^{4}$ vectors with weight 4, ⋯, $C_{N}^{\frac{N-5}{2}}$ vectors with weight $\frac{N-5}{2}$, and $C_{N-1}^{\frac{N-3}{2}}$ vectors (with the first component equal to 1) with weight $1+\frac{N-3}{2}$. The above vectors are denoted by $b_{1},b_{2},b_{3},\ldots ,b_{K}$, where $K=1+C_{N}^{2}+C_{N}^{4}+\cdots +C_{N}^{\frac{N-5}{2}}+C_{N-1}^{\frac{N-3}{2}}$. Let $A_{i}=b_{i}⊕\left(2\right)$ for 1≤i≤K. Take $A=\left(\begin{array}{c} A_{1}\\ A_{2}\\ \vdots \\ A_{K} \end{array}\right)$. Then $A_{i}$ and *A* are strength 1 orthogonal arrays and MD(A)=2. By Connection 1, $\left\{A_{1},A_{2},\ldots ,A_{K}\right\}$ can generate *K* one-uniform states, which form an orthogonal basis of a subspace *Q* of $C^{2⊗N}$. By Lemma 3, *Q* is an ((N,K,2)) QECC;

(2) By arguments similar to those used in the proof of (1), we can obtain the desired QECC. □

**Theorem** **3.** *Let L be an OA(r,N,2,2) with MD(L)≥3. If there exist vectors $b_{1},b_{2},\ldots ,b_{K}$ in $Z_{2}^{N}$ satisfying $\mathrm{HD}(b_{i},b_{j})\geq 3$ and $|\mathrm{HD}\left(b_{i},b_{j}\right)-\mathrm{HD}\left(L\right)|\geq 3$ for i≠j, then there is an ((N,K,3)) QECC.*

**Proof.**  Let $M_{i}=1_{r}⊗b_{i}+L$ for 1≤i≤K. Take $M=\left(\begin{array}{c} M_{1}\\ M_{2}\\ \vdots \\ M_{K} \end{array}\right)$. Both *M* and $M_{i}$ are OAs of strength two. Any two rows of *M* can be written as $m_{1}=b_{i}+l_{1},m_{2}=b_{j}+l_{2}$, where $b_{i},b_{j}\in \left\{b_{1},b_{2},\ldots ,b_{K}\right\}$, $l_{1},l_{2}\in L$.

(1) When i=j, $l_{1}\neq l_{2}$, $\mathrm{HD}\left(m_{1},m_{2}\right)=\mathrm{MD}\left(L\right)\geq 3$;

(2) When i≠j, $l_{1}=l_{2}$, $\mathrm{HD}\left(m_{1},m_{2}\right)=\mathrm{HD}\left(b_{i},b_{j}\right)\geq 3$;

(3) When i≠j and $l_{1}\neq l_{2}$, we have $\mathrm{HD}\left(m_{1},m_{2}\right)\geq \mathrm{HD}\left(b_{i}+l_{2},m_{2}\right)-\mathrm{HD}\left(b_{i}+l_{2},m_{1}\right)$ or $\mathrm{HD}\left(m_{1},m_{2}\right)\geq \mathrm{HD}\left(b_{i}+l_{2},m_{1}\right)-\mathrm{HD}\left(b_{i}+l_{2},m_{2}\right)$, so $\mathrm{HD}\left(m_{1},m_{2}\right)\geq \left|\mathrm{HD}\left(b_{i},b_{j}\right)-\mathrm{HD}\left(L\right)\right|\geq 3.$

So MD(M)≥3. By Connection 1, $\left\{M_{1},M_{2},\ldots ,M_{K}\right\}$ can generate *K* states, which form an orthogonal basis of a subspace *Q* of $C^{2⊗N}$. By Lemma 3, *Q* is an ((N,K,3)) QECC. □

**Theorem** **4.** *There exists a $\left(\left(3p,2^{p-n},3\right)\right)$ QECC with $2^{n-1}\leq p\leq 2^{n}-1$ for n≥3. In particular, for n=2, we have a ((9,2,3)) code.*

**Proof.**  Let $D=D\left(4,3,2\right)=\left(\begin{array}{ccc} 0 & 0 & 0\\ 0 & 0 & 1\\ 0 & 1 & 0\\ 0 & 1 & 1 \end{array}\right)$ be a difference scheme of strength 2. Take $L_{0}=\left(\left(2\right)⊗1_{2^{n-1}},1_{2}⊗\left(2\right)⊗1_{2^{n-2}},\ldots ,1_{2^{n-1}}⊗\left(2\right),L^{\prime }\right)$ is an OA$\left(2^{n},p,2,2\right)$ for $2^{n-1}\leq p\leq 2^{n}-1$ with n≥3 and $L_{i}=\left(\left(2\right)⊗1_{2^{n-1}},1_{2}⊗\left(2\right)⊗1_{2^{n-2}},\ldots ,1_{2^{n-1}}⊗\left(2\right),L^{\prime }+\left(1_{2^{n}}⊗R_{i}\right)\right)$ where $R_{i}$ is the *i*th row of $Z_{2}^{p-n}$ for $i=1,2,3,\ldots ,2^{p-n}$. Then $\left\{L_{1},L_{2},\ldots ,L_{2^{p-n}}\right\}$ is an orthogonal partition of strength 2 of $Z_{2}^{p}$. Let

$$M=\left(\begin{array}{c} D⊕L_{1}\\ D⊕L_{2}\\ \vdots \\ D⊕L_{2^{p-n}} \end{array}\right)=\left(\begin{array}{c} M_{1}\\ M_{2}\\ \vdots \\ M_{2^{p-n}} \end{array}\right),$$

By Lemma 4, $M_{i}=D⊕L_{i}$ is an OA of strength 2. Any two rows of $M_{i}$ can be written as $m_{1}=d_{1}⊕l_{1},m_{2}=d_{2}⊕l_{2}$, where $d_{1},d_{2}\in D$, $l_{1},l_{2}\in L_{i}$.

(1) When $d_{1}=d_{2}$, $\mathrm{HD}\left(m_{1},m_{2}\right)=3\cdot \mathrm{HD}\left(l_{1},l_{2}\right)\geq 3$;

(2) When $l_{1}=l_{2}$, $\mathrm{HD}\left(m_{1},m_{2}\right)=p\cdot \mathrm{HD}\left(d_{1},d_{2}\right)\geq 3$;

(3) When $d_{1}\neq d_{2}$ and $l_{1}\neq l_{2}$, we have

$$\mathrm{HD}\left(m_{1},m_{2}\right)=\left(3-\mathrm{HD}\left(d_{1},d_{2}\right)\right)\cdot \mathrm{HD}\left(l_{1},l_{2}\right)+\left(p-\mathrm{HD}\left(l_{1},l_{2}\right)\right)\cdot \mathrm{HD}\left(d_{1},d_{2}\right)\geq 3.$$

So $\mathrm{MD}(M_{i})\geq 3$.

Since *M* can be written as $D\left(4,3,2\right)⊕Z_{2}^{p}$ after row permutation, *M* is an OA of strength 2. Similarly, we also have MD(M)≥3. By Connection 1, $\left\{M_{1},M_{2},\ldots ,M_{2^{p-n}}\right\}$ can generate $2^{p-n}$ states, which form an orthogonal basis of a subspace *Q* of $C^{2⊗3p}$. By Lemma 3, *Q* is a $\left(\left(3p,2^{p-n},3\right)\right)$ QECC.

Especially, when n=2 and p=3, a ((9,2,3)) QECC exists with logical codewords: $|\varphi_{1}\rangle =\frac{1}{4}(|000000000\rangle +|011011011\rangle +|101101101\rangle +|110110110\rangle +|000000111\rangle +|011011100\rangle +|101101010\rangle +|110110001\rangle +|000111000\rangle +|011100011\rangle +|101010101\rangle +|110001110\rangle +|000111111\rangle +|011100100\rangle +|101010010\rangle +\left|110001001\rangle \right)$, $|\varphi_{2}\rangle =\frac{1}{4}(|001001001\rangle +|010010010\rangle +|100100100\rangle +|111111111\rangle +|001001110\rangle +|010010101\rangle +|100100011\rangle +|111111000\rangle +|001110001\rangle +|010101010\rangle +|100011100\rangle +|111000111\rangle +|001110110\rangle +|010101101\rangle +|100011011\rangle +\left|111000000\rangle \right)$.

The code is pure, but neither the 9 qubit Shor code in [1] nor the 9 qubit Ruskai code in [11] are pure. □

**Theorem** **5.** *There exists a $\left(\left(4p,2^{p-n+1},3\right)\right)$ QECC with $2^{n-1}\leq p\leq 2^{n}-1$ for n≥3. In particular, for n=2, we have a ((12,4,3)) code.*

**Proof.**  Take $D_{0}=D\left(4,4,2\right)=\left(\begin{array}{cccc} 0 & 0 & 0 & 0\\ 0 & 0 & 1 & 1\\ 0 & 1 & 0 & 1\\ 0 & 1 & 1 & 0 \end{array}\right)$ and $D_{1}=D\left(4,4,2\right)=\left(\begin{array}{cccc} 0 & 0 & 0 & 1\\ 0 & 0 & 1 & 0\\ 0 & 1 & 0 & 0\\ 0 & 1 & 1 & 1 \end{array}\right)$. Then $\left\{D_{0},D_{1}\right\}$ is a partition of strength 2 of the difference scheme $D\left(8,4,2\right)=\left(0_{8},Z_{2}^{3}\right)$. For $2^{n-1}\leq p\leq 2^{n}-1$ and n≥3, let

$$M=\left(\begin{array}{c} D_{0}⊕L_{1}\\ \vdots \\ D_{0}⊕L_{2^{p-n}}\\ D_{1}⊕L_{1}\\ \vdots \\ D_{1}⊕L_{2^{p-n}} \end{array}\right)=\left(\begin{array}{c} M_{1}\\ \vdots \\ M_{2^{p-n}}\\ M_{2^{p-n}+1}\\ \vdots \\ M_{2^{p-n+1}} \end{array}\right),$$

where $L_{1},L_{2},\ldots ,L_{2^{p-n}}$ are as in Theorem 5. Similar arguments in Theorem 2 apply to *M*, we can obtain the desired QECCs.

Especially, when n=2 and p=3, a ((12,4,3)) code can be attained. □

**Theorem** **6.** *There exists a $\left(\left(4p,2^{p-n+1},4\right)\right)$ QECC with $2^{n-2}+1\leq p\leq 2^{n-1}$ for n≥4. In particular, for n=3, we have a ((16,4,4)) code.*

**Proof.**  Let $D_{0}=D_{3}\left(4,4,2\right)=\left(\begin{array}{cccc} 0 & 0 & 0 & 0\\ 0 & 0 & 1 & 1\\ 0 & 1 & 0 & 1\\ 0 & 1 & 1 & 0 \end{array}\right)$ and $D_{1}=D_{3}\left(4,4,2\right)=\left(\begin{array}{cccc} 0 & 0 & 0 & 1\\ 0 & 0 & 1 & 0\\ 0 & 1 & 0 & 0\\ 0 & 1 & 1 & 1 \end{array}\right)$. Then $\left\{D_{0},D_{1}\right\}$ is a partition of strength 2 of the difference scheme $D\left(8,4,2\right)=\left(0_{8},Z_{2}^{3}\right)$. Take $L_{0}=\left(\left(2\right)⊗1_{2^{n-1}},1_{2}⊗\left(2\right)⊗1_{2^{n-2}},\ldots ,1_{2^{n-1}}⊗\left(2\right),L^{\prime }\right)$ is an OA$\left(2^{n},p,2,3\right)$ for $2^{n-2}+1\leq p\leq 2^{n-1}$ with n≥4 and $L_{i}=\left(\left(2\right)⊗1_{2^{n-1}},1_{2}⊗\left(2\right)⊗1_{2^{n-2}},\ldots ,1_{2^{n-1}}⊗\left(2\right),L^{\prime }+\left(1_{2^{n}}⊗R_{i}\right)\right)$ where $R_{i}$ is the *i*th row of $Z_{2}^{p-n}$ for $i=1,2,3,\ldots ,2^{p-n}$. Then $\left\{L_{1},L_{2},\ldots ,L_{2^{p-n}}\right\}$ is an orthogonal partition of strength 3 of $Z_{2}^{p}$. Let

$$M=\left(\begin{array}{c} D_{0}⊕L_{1}\\ \vdots \\ D_{0}⊕L_{2^{p-n}}\\ D_{1}⊕L_{1}\\ \vdots \\ D_{1}⊕L_{2^{p-n}} \end{array}\right)=\left(\begin{array}{c} M_{1}\\ \vdots \\ M_{2^{p-n}}\\ M_{2^{p-n}+1}\\ \vdots \\ M_{2^{p-n+1}} \end{array}\right),$$

Similar arguments in Theorem 5 apply to *M*, we can obtain the desired QECCs.

Especially, when n=3 and p=4, a ((16,4,4)) code exists. □

**Theorem** **7.** *Suppose $L^{N}$ denotes an OA(r,N,2,t). Let $Y=(0_{2}⊕L^{N_{1}},\left(2\right)⊕L^{N-N_{1}})$. If MD(Y)≥t+1, then an ((N,2,t+1)) QECC exists.*

**Proof.**  Let $Y_{i}=\left(L^{N_{1}},i+L^{N-N_{1}}\right)$ for i=0,1. Thus $Y=\left(\begin{array}{c} Y_{0}\\ Y_{1} \end{array}\right)$. Obviously, $Y_{i}$ is an OA(r,N,2,t) and *Y* is an OA(2r,N,2,t). If MD(Y)≥t+1, then $\mathrm{MD}\left(Y_{i}\right)\geq \mathrm{MD}\left(Y\right)\geq t+1$. From Lemma 3, there exists an ((N,2,t+1)) QECC. □

**Theorem** **8.** *Let L be an OA(r,N,2,t) with MD(L)≥t+1. If there exist vectors $b_{1},b_{2},\ldots ,b_{K}$ in $Z_{2}^{N}$ such that $\mathrm{MD}\left(\begin{array}{c} 1_{r}⊗b_{1}+L\\ 1_{r}⊗b_{2}+L\\ \vdots \\ 1_{r}⊗b_{K}+L \end{array}\right)\geq t+1$, then there is an ((N,K,t+1)) QECC.*

**Proof.**  Let $M=\left(\begin{array}{c} M_{1}\\ M_{2}\\ \vdots \\ M_{K} \end{array}\right)=\left(\begin{array}{c} 1_{r}⊗b_{1}+L\\ 1_{r}⊗b_{2}+L\\ \vdots \\ 1_{r}⊗b_{K}+L \end{array}\right)$. Obviously, $M_{i}$ is an OA(r,N,2,t) and MD(M)≥t+1. From Lemma 3, there exists an ((N,K,t+1)) QECC. □

**Theorem** **9.** *There exists a $\left(\left(2\left(m_{d}+1\right)\left(d-1\right),1,d\right)\right)$ QECC for any integer d≥5, where $m_{d}$ is the integer that satisfies $2^{m_{d}-1}+2\leq d\leq 2^{m_{d}}+1$. Especially, for d=3,4, we have three QECCs ((6,1,3)), ((8,1,4)) and ((10,1,4)).*

**Proof.**  Let $s=2^{m_{d}+1}$. From Lemma 6, an OA$\left(s^{d-1},s+1,s,d-1\right)$ exists. Obviously, s+1≥2d, then an OA$\left(s^{d-1},2\left(d-1\right),s,d-1\right)$ exists and is denoted by *A*. From Lemma 2, MD(A)=d. Replacing the *s* levels, 0,1,…,s−1, by distinct rows of $Z_{2}^{m_{d}+1}$ respectively, we can get an IrOA$\left(2^{\left(m_{d}+1\right)\left(d-1\right)},2\left(d-1\right)\left(m_{d}+1\right),2,d-1\right)$. By Lemma 3, a $\left(\left(2\left(d-1\right)\left(m_{d}+1\right),1,d\right)\right)$ QECC exists.

Especially, when d=3,4, by using Lemma 3 and IrOA(8,6,2,2), IrOA(16,8,2,3), and IrOA(24,10,2,3), three QECCs ((6,1,3)), ((8,1,4)), ((10,1,4)) can be obtained. □

**Corollary** **1.** *For any d≥5, let $m_{d}$ be the integer satisfying $2^{m_{d}-1}+2\leq d\leq 2^{m_{d}}$. Then an $\left(\left(n_{d},1,d\right)\right)$ QECC exists for $2\left(d-1\right)\left(m_{d}+1\right)\leq n_{d}\leq 2d\left(m_{d}+1\right)-1$. In particular, a QECC $\left(\left(n_{d}^{\prime },1,2^{m_{d}}+1\right)\right)$ exists for $\left(2^{m_{d}+1}\right)\left(m_{d}+1\right)\leq n_{d}^{\prime }\leq \left(2^{m_{d}+1}+1\right)\left(m_{d}+1\right)$.*

**Proof.**  Let $s=2^{m_{d}+1}$. From Lemma 6, an OA$\left(s^{d-1},s+1,s,d-1\right)$ exists. Obviously, *B*=OA$(s^{d-1}$, 2d,s,d−1) exists since s+1≥2d. From Lemma 2, MD(B)=d+2. By using the replacement method in Theorem 9, we can get *C*=OA$\left(s^{d-1},2d\left(m_{d}+1\right),2,d-1\right)$. Removing the last $1,2,\ldots ,2m_{d}+2$ columns from *C*, we can get an OA$\left(s^{d-1},n_{d},2,d-1\right)$ with MD≥d for $2\left(d-1\right)\left(m_{d}+1\right)\leq n_{d}\leq 2d\left(m_{d}+1\right)-1$. By Lemma 3, the desired $\left(\left(n_{d},1,d\right)\right)$ QECC exists.

Similarly, from the OA$\left(s^{d-1},s+1,s,d-1\right)$, we can obtain an OA$\left(s^{d-1},\left(s+1\right)\left(m_{d}+1\right),2,d-1\right)$. Then removing the last $0,1,\ldots ,m_{d}+1$ columns, we can have the desired result by Lemma 3. □

## 4. Examples

In this section, we use examples to illustrate applications of theorems.

**Example** **1.** *Construction of a ((4,K,2)) QECC for any integer 1≤K≤4.*

*Let t=3 in Theorem 1. Take $D_{3}\left(4,4,2\right)=\left(\begin{array}{c} d_{1}\\ d_{2}\\ d_{3}\\ d_{4} \end{array}\right)=\left(\begin{array}{cccc} 0 & 0 & 0 & 1\\ 0 & 0 & 1 & 0\\ 0 & 1 & 0 & 0\\ 0 & 1 & 1 & 1 \end{array}\right)$, $A=D_{3}\left(4,4,2\right)⊕\left(2\right)$ and $A_{i}=d_{i}⊕\left(2\right)$ for 1≤i≤4. Then $A_{i}$(1≤i≤4) can produce four states, $\varphi_{1}=\frac{1}{\sqrt{2}}(|0001\rangle +\left|1110\rangle \right)$, $\varphi_{2}=\frac{1}{\sqrt{2}}(|0010\rangle +\left|1101\rangle \right)$, $\varphi_{3}=\frac{1}{\sqrt{2}}(|0100\rangle +\left|1011\rangle \right)$, $\varphi_{4}=\frac{1}{\sqrt{2}}(|0111\rangle +\left|1000\rangle \right)$, which form an orthogonal basis of a subspace Q in $C^{2⊗4}$. Therefore, Q is an optimal ((4,4,2)) QECC which can be found in [7].*

*Furthermore, if taking $Q_{K}$ to be the subspace spanned by $\left\{\varphi_{1},\ldots ,\varphi_{K}\right\}$ for 1≤K≤3, then we obtain a ((4,K,2)) QECC.*

The QECCs in Example 1 are different from and particularly when K=1,2, have less number of items for every basis state than those codes in [12]. To be self-contained, the ((4,K,2)) QECCs for K=1,2,4 in [12] are provided as follows.

((4,1,2)): $|\phi \rangle =\frac{1}{2}(|0000\rangle +|1100\rangle +|0011\rangle +\left|1111\rangle \right)$.

((4,2,2)): $|\phi_{1}\rangle =\frac{1}{2}(|0000\rangle +|1010\rangle +|0101\rangle +\left|1111\rangle \right)$, $|\phi_{2}\rangle =\frac{1}{2}(|0011\rangle +|1001\rangle +|0110\rangle +\left|1100\rangle \right)$.

((4,4,2)): $|\phi_{1}\rangle =\frac{1}{\sqrt{2}}(|0000\rangle +\left|1111\rangle \right)$, $|\phi_{2}\rangle =\frac{1}{\sqrt{2}}(|0011\rangle +\left|1100\rangle \right)$, $|\phi_{3}\rangle =\frac{1}{\sqrt{2}}(|1010\rangle +\left|0101\rangle \right)$, $|\phi_{4}\rangle =\frac{1}{\sqrt{2}}(|0110\rangle +\left|1001\rangle \right)$.

Comparison of the method of code construction with [7].

Both methods can take any classical code to a quantum code. The method proposed in [7] can make it by solving for the amplitudes in the superposition. Since any classical code $\left(N,m,d^{\prime }\right)$ is an OA(m,N,2,t), the method in this paper can produce a quantum code $\left(\left(N,1,d^{\prime \prime }\right)\right)$ which is also a $\left(d^{\prime \prime }-1\right)$-uniform state where $d^{\prime \prime }=min\left\{d^{\prime },t+1\right\}$ from Connection 1. Moreover, if the OA(m,N,2,t) with an orthogonal partition $\left\{A_{1},A_{2},\ldots ,A_{K}\right\}$ of strength $t_{1}$, this method can produce a quantum code ((N,K,d)) where $d=min\{d^{\prime },t_{1}+1\}$. The amplitudes in the superposition for each logical codeword are all equal to $\sqrt{\frac{m}{K}}$. For example, the code ((4,4,2)) in Example 1 after it is normalized is the same as the one constructed using the method proposed in [7]. It is noteworthy that in Example 1 if taking $D=\left(\begin{array}{cccc} 0 & 0 & 0 & 0\\ 0 & 0 & 1 & 1\\ 0 & 1 & 0 & 1\\ 0 & 1 & 1 & 0 \end{array}\right)$, then we can construct a stabilizer code with parameter ((4,4,2)) whose logical codewords are $\varphi_{1}=\frac{1}{\sqrt{2}}(|0000\rangle +\left|1111\rangle \right)$, $\varphi_{2}=\frac{1}{\sqrt{2}}(|0011\rangle +\left|1100\rangle \right)$, $\varphi_{3}=\frac{1}{\sqrt{2}}(|0101\rangle +\left|1010\rangle \right)$, $\varphi_{4}=\frac{1}{\sqrt{2}}(|0110\rangle +\left|1001\rangle \right)$.

**Example** **2.** *(1) For N=5, take $b_{1}=\left(00000\right)$, $b_{2}=\left(11000\right)$, $b_{3}=\left(10100\right)$, $b_{4}=\left(10010\right)$, and $b_{5}=\left(10001\right)$. Let $A_{i}=b_{i}⊕\left(2\right)$ for 1≤i≤5. Then $A_{i}$(1≤i≤5) can produce five states. By Theorem 2, Q is a ((5,5,2)) QECC;*

*(2) For N=7, take $b_{1}=\left(0000000\right)$, $b_{2}=\left(0000011\right)$, $b_{3}=\left(0000101\right)$, $b_{4}=\left(0000110\right)$, $b_{5}=\left(0001001\right)$, $b_{6}=\left(0001010\right)$, $b_{7}=\left(0001100\right)$, $b_{8}=\left(0010001\right)$, $b_{9}=\left(0010010\right)$, $b_{10}=\left(0010100\right)$, $b_{11}=\left(0011000\right)$, $b_{12}=\left(0100001\right)$, $b_{13}=\left(0100010\right)$, $b_{14}=\left(0100100\right)$, $b_{15}=\left(0101000\right)$, $b_{16}=\left(0110000\right)$, $b_{17}=\left(1000001\right)$, $b_{18}=\left(1000010\right)$, $b_{19}=\left(1000100\right)$, $b_{20}=\left(1001000\right)$, $b_{21}=\left(1010000\right)$, $b_{22}=\left(1100000\right)$. Let $A_{i}=b_{i}⊕\left(2\right)$. Then $A_{i}$(1≤i≤22) can produce 22 states. With Theorem 2, they yield a ((7,22,2)) QECC.*

**Example** **3.** *Construction of a ((7,2,3)) QECC.*

*Let r=8 and N=7 in Theorem 3. The two vectors $b_{1}=\left(0000000\right)$ and $b_{2}=\left(1111111\right)$ can be used to construct a ((7,2,3)) QECC whose basis states are:*

*$|\varphi_{1}\rangle =\frac{1}{2\sqrt{2}}(|0000000\rangle +|0010111\rangle +|0101011\rangle +|0111100\rangle +|1001101\rangle +|1011010\rangle +|1100110\rangle +\left|1110001\rangle \right)$ and*

*$|\varphi_{2}\rangle =\frac{1}{2\sqrt{2}}(|1111111\rangle +|1101000\rangle +|1010100\rangle +|1000011\rangle +|0110010\rangle +|0100101\rangle +|0011001\rangle +\left|0001110\rangle \right)$.*

*This is in fact equivalent to the Steane code. It can correct one error such as $e=I_{2}⊗I_{2}⊗I_{2}⊗I_{2}⊗I_{2}⊗I_{2}⊗I_{2}⊗\sigma_{x}$, $I_{2}⊗I_{2}⊗I_{2}⊗I_{2}⊗I_{2}⊗I_{2}⊗\sigma_{y}⊗I_{2}$ and so on.*

**Example** **4.** *Construction of a $\left(\left(3p,2^{p-n},3\right)\right)$ QECC with $2^{n-1}\leq p\leq 2^{n}-1$ for n=3,4.*

*(1) Let n=3, p=4,5,6,7 in Theorem 4. We can obtain QECCs ((12,2,3)), ((15,4,3)), ((18,8,3)), ((21,16,3));*

*(2) Let n=4, p=8,9,…,15 in Theorem 4. One gets QECCs ((24,16,3)), ((27,32,3)), …, $\left(\left(45,2^{11},3\right)\right)$.*

**Example** **5.** *Construction of a $\left(\left(4p,2^{p-n+1},4\right)\right)$ QECC with $2^{n-2}+1\leq p\leq 2^{n-1}$ for n=4,5.*

*For the case n=4 and $2^{2}+1\leq p\leq 2^{3}$, Theorem 6 produces QECCs ((20,4,4)), ((24,8,4)), ((28,16,4)), ((32,32,4)).*

*For the case n=5 and $2^{3}+1\leq p\leq 2^{4}$, Theorem 6 yields QECCs ((36,32,3)), ((40,64,3)), …, $\left(\left(64,2^{12},4\right)\right)$.*

**Example** **6.** *For N=23 and $N_{1}=16$, take $L^{23}=\left(a_{1},\ldots ,a_{23}\right)$ to be the OA(2048,23,2,6) (the first 2048 runs and the first 23 columns from OA(4096,24,2,7) in [45]). Let $L^{16}=\left(a_{1},a_{2},\ldots ,a_{16}\right)$ and $L^{7}=\left(a_{17},a_{18},\ldots ,a_{23}\right)$. Then MD(Y)=7. Theorem 7 yields a ((23,2,7)) QECC.*

**Example** **7.** *For r=512 and N=23, take L to be the OA(512,23,2,4) (the first 512 runs and the first 23 columns from OA(1024,24,2,5) in [45]). We can get $b_{1},b_{2},\ldots ,b_{9}\in Z_{2}^{23}$ that satisfies the conditions in Theorem 8 where $b_{1}=\left(00000000000000000000000\right)$, $b_{2}=\left(11111111111111111111111\right)$, $b_{3}=\left(00000000000000000111011\right)$, $b_{4}=\left(00000000000000011011101\right)$, $b_{5}=\left(00000000000001010000111\right)$, $b_{6}=\left(00000000000001101001011\right)$, $b_{7}=\left(00000000000011110011110\right)$, $b_{8}=\left(00000000000110010001010\right)$, $b_{9}=\left(00000000001100110111110\right)$. Then we can construct a ((23,9,5)) QECC.*

**Example** **8.** *Comparison of the ((10,1,4)) QECCs in Theorem 9,^12^,^46^].*

*The new quantum state in the QECC ((10,1,4)) in Theorem 9 has 24 terms. The quantum state in the QECC ((10,1,4)) in [12] has 1024 terms. The quantum state in the QECC ((10,1,4)) in [46] with the follow stablizer matrix G has 512 terms where*

$$G=\left(\begin{array}{cc} 1100110000 & |\;1111110000\\ 0111111000 & |\;0111111000\\ 0011001100 & |\;0011111100\\ 0001111110 & |\;0001111110\\ 0000110011 & |\;0000111111\\ 1111110000 & |\;0011000000\\ 0111111000 & |\;0001100000\\ 0011111100 & |\;0000110000\\ 0001111110 & |\;0000011000\\ 0000111111 & |\;0000001100 \end{array}\right).$$

*Compared with the above two codes, it is clear that our construction method has the advantage of a small number of terms.*

**Example** **9.** *Some new QECCs with larger minimum distance by Corollary 1.*

*Let d=94. Then $m_{d}=7$ and we have an $\left(\left(n_{d},1,94\right)\right)$ QECC for $1488\leq n_{d}\leq 1503$.*

*Let d=66. Then $m_{d}=7$ and we have an $\left(\left(n_{d},1,66\right)\right)$ QECC for $1040\leq n_{d}\leq 1055$.*

*Let d=41. Then $m_{d}=6$ and we have an $\left(\left(n_{d},1,41\right)\right)$ QECC for $560\leq n_{d}\leq 573$.*

*Let d=23. Then $m_{d}=5$ and we have an $\left(\left(n_{d},1,23\right)\right)$ QECC for $264\leq n_{d}\leq 275$.*

*Let d=129. Then $m_{d}=7$ and we have an $\left(\left(n_{d}^{\prime },1,129\right)\right)$ QECC for $2048\leq n_{d}^{\prime }\leq 2056$.*

*Let d=33. Then $m_{d}=5$ and we have an $\left(\left(n_{d}^{\prime },1,33\right)\right)$ QECC for $384\leq n_{d}^{\prime }\leq 390$.*

## 5. Conclusions

In the work, by using OAs, we study the relation between uniform states and binary QECCs. Several methods for constructing QECCs from OAs are presented. Some optimal QECCs are obtained. Our methods have three advantages. The first is to be able to construct an $\left(\left(N,K_{1},d\right)\right)$ QECC from each ((N,K,d)) QECC we construct for arbitrary integer $1\leq K_{1}\leq K$. The second is that Theorems 1 and 7–9 can be generalized to construct QECCs ${\left(\left(N,K,d\right)\right)}_{q}$ for arbitrary *d* and a prime power *q*. The third is that for the constructed QECCs, their every basis state has less than or equal to terms compared with the existing binary QECCs in [12,41]. A link between an IrOA and the uniform state is established by Connection 1. In fact, from Theorem 1 to Theorem 9 we always make quantum codes by using uniform states generated by orthogonal partitions. On the other hand, when a quantum code is pure we can easily obtain uniform states. For example, each of the logical codewords in the quantum code ((4,4,2)) in [7] is a one-uniform state. When it is not pure it is worth studying how to use quantum codes to make uniform states. In the future, we will also investigate constructing more optimal QECCs with d>2.

## Figures and Tables

**Table 1:** Correspondence between parameters of OAs and QECCs.

	OAs	QECCs
*N*	Number of factors	Length of code
*K*	Number of partitioned blocks	Dimension of code
*d*	min$\left\{t_{1}+1,\mathrm{MD}\left(A\right)\right\}$	MD of code
*s*	Number of levels	alphabet size

**Table 2:** Comparison of the obtained QECCs with those in [12].

	The QECCs in [12]			The QECCs by Theorem 1		
	((4,K,2))	((6,K,2))	((8,K,2))	((4,K,2))	((6,K,2))	((8,K,2))
*K*	1, 2, 4	$2^{2}$, $2^{3}$, $2^{4}$	$2^{4}$, $2^{5}$, $2^{6}$	1, 2, 3, 4	1, 2, 3, *…*, $2^{4}$	1, 2, 3, *…*, $2^{6}$
No.	4, 4, 2	8, 4, 2	8, 4, 2	2, 2, 2, 2	2, 2, 2, *…*,2	2, 2, 2, *…*, 2

## Data Availability

Not applicable.
